# Transcatheter Arterial Embolization for Blunt Splenic Injury With Resuscitative Endovascular Balloon Occlusion of the Aorta: The Significance of Early Involvement of Radiologists

**DOI:** 10.7759/cureus.53753

**Published:** 2024-02-07

**Authors:** Shinji Wada, Junichi Matsumoto, Masaya Osugi, Keisuke Ida, Hidefumi Mimura

**Affiliations:** 1 Department of Diagnostic and Interventional Radiology, St. Marianna University School of Medicine, Kawasaki, JPN; 2 Department of Emergency and Critical Care Medicine, St. Marianna University School of Medicine, Kawasaki, JPN; 3 Division of Gastroenterological and General Surgery, St. Marianna University School of Medicine, Kawasaki, JPN

**Keywords:** transcatheter arterial embolization, splenic injury, splenic artery embolization, resuscitative endovascular balloon occlusion of the aorta, interventional radiology, hemodynamic instability

## Abstract

Splenectomy is a common procedure for managing splenic injury in patients with unstable vital signs. Transcatheter arterial embolization (TAE) has emerged as a limited alternative to splenectomy, although the role of TAE can be expanded upon the stabilization of vital signs. The current case report discusses a man in his 50s, in shock after a motor vehicle accident, who was successfully stabilized using resuscitative endovascular balloon occlusion of the aorta (REBOA), followed by splenic artery embolization (SAE) instead of splenectomy, with early involvement of diagnostic and interventional radiologists from the initial stage of care. We also discuss the difficulties of SAE under REBOA and the significance of the early involvement of radiologists in trauma care.

## Introduction

Splenectomy is frequently performed in cases of splenic injury with unstable vital signs. Transcatheter arterial embolization (TAE) is a key, less invasive treatment modality [1]. However, the application of TAE in splenic injury can be expanded when vital signs are stabilized. Resuscitative endovascular balloon occlusion of the aorta (REBOA), used to achieve aortic occlusion percutaneously, can be effectively employed for endovascular management in cases of hemorrhagic shock [2]. Theoretically, indications for TAE should be expanded if REBOA can stabilize vital signs; however, there are limited reports using TAE, particularly splenic artery embolization (SAE), as an alternative to splenectomy in trauma patients with splenic injury. Current data favor the use of proximal and coil embolization techniques in adults; distal embolization may be beneficial in patients with focal, angiographically evident injury to preserve access for future intervention and minimize global splenic ischemia [3].

Herein, we report a case in which diagnostic and interventional radiologists were involved from the initial resuscitative phase, and in which the use of REBOA stabilized hemodynamics and broadened the indications for TAE. We also discuss how TAE under REBOA is performed under conditions that differ from physiologic circulation, complicating the TAE endpoint.

## Case presentation

**Figure 1:**
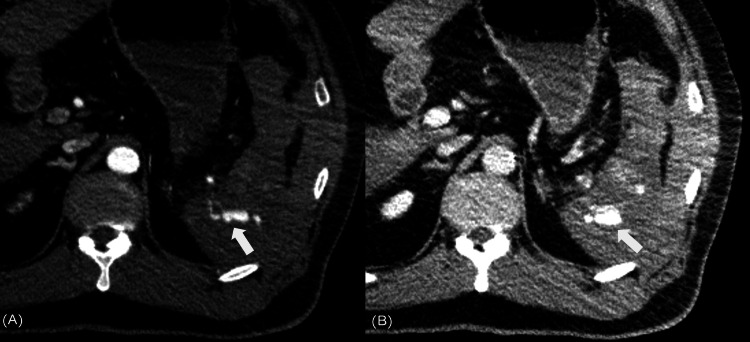
Whole body CT performed immediately before REBOA balloon deflated (A) Contrast-enhanced computed tomography arterial phase indicating extravasation (arrow) outside the spleen. (B) In the venous phase, extravasation (arrow) appears enlarged, indicating active bleeding. CT, computed tomography; REBOA, resuscitative endovascular balloon occlusion of the aorta

A man in his 50s, 173 cm in height, weighing 103 kg, had been in a motor vehicle accident at approximately 10:00 pm. He was transported to the emergency room ~40 min later. Blood pressure was not measurable during admission, and focused assessment with sonography in trauma revealed fluid collection around the spleen. Fluid and blood transfusions were initiated, and a 7-Fr intra-aortic balloon occlusion catheter (RESCUE BALLOON, Tokai Medical Products, Aichi, Japan) was inserted through the right common femoral artery, with a 5-Fr sheath inserted through the left common femoral artery by the on-duty radiologist. In the trauma bay, the REBOA catheter was inflated in the thoracic part of the descending aorta to stabilize the hemodynamic status after a plain chest radiograph excluded chest injury. The interventional radiologist convened at the time of patient acceptance arrived at the hospital, and the balloon was slowly deflated immediately before the computed tomography (CT) scan, ensuring stable vital signs, and a whole-body CT was performed. During the CT examination, the diagnostic and interventional radiologists read the scan and diagnosed the patient with splenic injury with extravasation and laceration (equivalent to the American Association for the Surgery of Trauma-organ injury scale grade IV) [4]. After confirming the absence of injury in other organs, the decision was made to perform TAE after on-site consultation with the surgeon and interventional radiologist (Figure 1).

**Figure 2:**
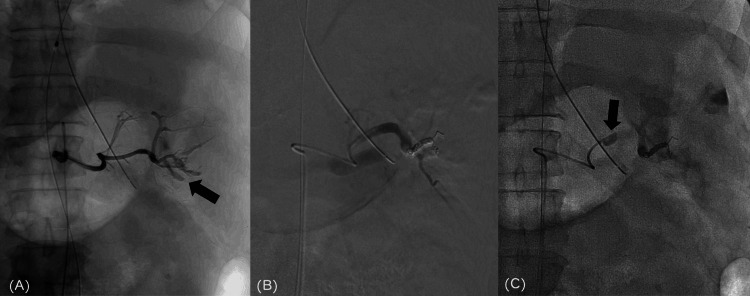
Distal splenic artery embolization and a balloon catheter placement in the middle part of the splenic artery (A) Extravasation (arrow) from the inferior pole branch of the splenic artery is noted. (B) The lower pole branch was embolized with pushable coils, and the upper pole branch was embolized with a gelatin sponge; however, extravasation from the latter was unknown. (C) Extravasation from the splenic artery dissipated, but blood pressure failed to increase. Therefore, a balloon catheter (arrow) was placed in the middle part of the splenic artery, inflated, and the patient was returned to the intensive care unit.

Upon transfer to the angiography suite, angiography was initiated with the assistance of REBOA, considering the vital signs of the patient. When a systolic blood pressure of about 80 mmHg could be maintained, the REBOA was half deflated, and when the systolic blood pressure was less than 80 mmHg, it was fully inflated. The catheter was inserted into the left common femoral artery via a sheath introducer, and celiac arteriography revealed extravasation in the superior pole branch of the splenic artery. Although laboratory data at emergency room arrival showed no significant coagulopathy, with a fibrinogen level of 198 mg/dl and platelet count of 231 × 10^3^/μl, immediate embolization was considered appropriate. Embolization of the upper and lower pole branches of the splenic artery was performed using a gelatin sponge (Serescue, Astellas Pharma, Tokyo, Japan). The splenic lower pole artery, considered the main bleeding source, was embolized using 0.016- and 0.018-inch pushable coils (C-STOPPER COIL, PIOLAX, Kanagawa, Japan; Tornade, COOK, Indiana, United States), dissipating extravasation; however, the blood pressure did not increase. During splenic arteriography, the REBOA was completely deflated, and the patient was managed with partial deflation while observing vital signs. Subsequently, a balloon catheter (Selecon MP Catheter II, TERUMO, Tokyo, Japan) was placed in the main splenic artery to eliminate the source of bleeding (Figure 2). The patient’s fibrinogen level and platelet count immediately before the first TAE had dropped to 156 mg/dl and 156 x 10^3^/µl, respectively.

**Figure 3:**
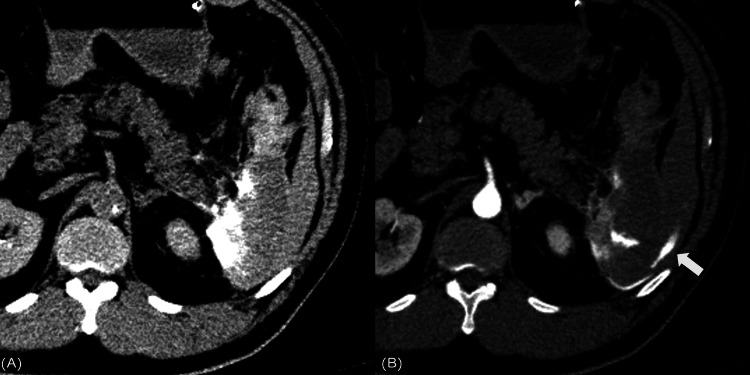
Second CT examination (A) Non contrast-enhanced CT after the first embolization indicating a peri-splenic contrast accumulation. (B) Subsequent contrast-enhanced CT arterial phase indicating extravasation (arrow). CT, computed tomography

On returning to the intensive care unit, transfusion was adjusted; however, it could not be ascertained whether hemostasis had been achieved clinically, documenting a systolic blood pressure of ~60 mmHg. Therefore, the splenic artery balloon was inflated, but REBOA was not used thereafter. The balloon catheter placed in the splenic artery was deflated immediately before the CT scan, and a transvenous contrast-enhanced CT scan was performed. Given the continued extravasation surrounding the spleen with no other sources of bleeding, we decided to perform another SAE (Figure 3).

**Figure 4:**
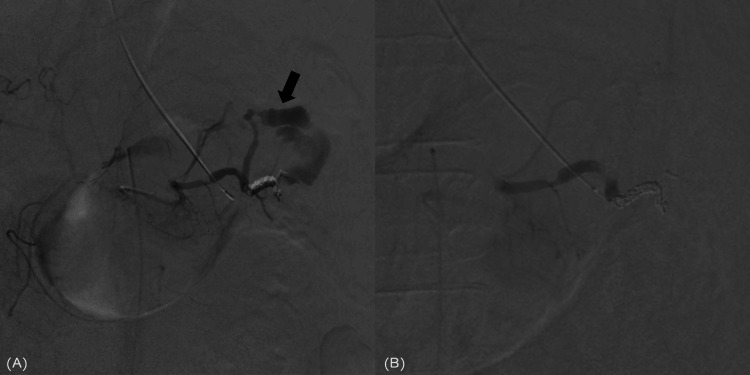
Second splenic artery embolization (A) A second splenic arteriography showed massive extravasation (arrow) from the superior pole branch. (B) The upper pole branch was embolized using 33% N-butyl cyanoacrylate, resulting in the dissipation of extravasation.

Subsequently, the patient was shifted back to the angiography suite. Owing to the recanalization of previously embolized arteries, another embolization procedure was performed using 33% N-butyl cyanoacrylate (NBCA) (Histoacryl, B BRAUN, Tokyo, Japan) to achieve complete hemostasis, with systolic blood pressure increasing to ~110 mmHg (Figure 4). The total transfusion volume comprised 3900 ml of concentrated red blood cells, 2400 ml of fresh frozen plasma, and 400 ml of platelet concentrate. The intermittent occlusion time of the aorta by REBOA was ~60 min, and the partial occlusion time was ~20 min.

Despite systemic management, temporary continuous renal replacement therapy was required owing to the development of renal failure, along with tracheotomy because of prolonged intubation. By day 50, renal function had normalized, the tracheostomy was closed on day 79, and the patient was discharged on day 95. A follow-up CT at 12 months revealed that approximately half of the spleen remained, and no complications were observed.

## Discussion

Herein, we present a unique case of splenic injury in which REBOA was employed as part of the initial treatment. The patient’s life was saved, and splenectomy was avoided, by the involvement of a diagnostic radiologist’s confirmation of the CT diagnosis, an interventional radiologist from the initial treatment, and subsequent TAE performed at the angiosite. To the best of our knowledge, this is the first case report documenting this specific approach.

The positive aspect of this case lies in the ability to stabilize the vital signs of a patient with trauma under hemorrhagic shock using REBOA, identify a single splenic injury using whole-body CT, and successfully manage the condition using SAE. Although whole-body CT allows appropriate management based on the trauma injury location and severity and provides detailed information necessary for interventional radiology, the more hemodynamically unstable the patient, the greater the need to develop a strategy capable of eliciting superior benefits in the least amount of time. The current case exemplifies the importance of time-sensitive endovascular management by promptly involving diagnostic and interventional radiologists from the primary survey, a very early phase of care [5].

REBOA serves as a viable alternative to resuscitative thoracotomy in cases of traumatic shock and is an effective bridge to definitive treatment while preserving blood flow to the brain and coronary arteries [6]. Two approaches for REBOA are available: partial REBOA, in which the balloon is partially inflated, and intermittent REBOA, involving alternating cycles of complete inflation and deflation [7,8]. The timing of balloon deflation helps perform SAE. However, the utilization of partial REBOA during SAE requires close monitoring and regulation by a trained physician, given the substantial hemodynamic impact and management complexities associated with even minimal changes in balloon volume.

According to the World Society of Emergency Surgery (WSES) classification and guidelines for splenic trauma, operative management is recommended for severe cases (WSES IV) with hemodynamic instability [1]. However, in a retrospective cohort study by Zoppo et al., no statistically significant differences in adverse events or 30-day survival were observed between 39 patients who were hemodynamically stable and 13 who were hemodynamically unstable after undergoing SAE [9]. Ogura et al. reported a trauma case series involving the use of TAE with REBOA, in which four out of seven cases presented with hemodynamically unstable splenic injuries, and all were successfully managed without additional surgical interventions [2]. Although surgical management is typically the standard approach for patients who are hemodynamically unstable, management strategies could be modified if the hemodynamic status can be stabilized using REBOA.

The first SAE dissipated extravasation, resulting in incomplete embolization. Angiography under REBOA assistance differs from physiologic blood flow and could complicate the establishment of the embolization endpoint. Considering the development of coagulopathy at the initial TAE and the possibility that the gelatin sponge was pushed away by increased blood flow due to improvement in shock, robust embolization with NBCA at the initial SAE would have been necessary. When vital signs fail to improve following SAE, as in the present case report, placement of a balloon catheter in the main trunk of the splenic artery, along with additional CT evaluation and careful consideration of immediate conversion to splenectomy, should be pursued. In a room equipped with CT and angiography, facilitating seamless diagnosis and treatment, CT diagnosis, interventional radiology, and surgery can be performed without patient transfer, circumventing the back-and-forth between CT and angiography rooms, as in the current case [10].

The development of acute kidney injury, documented in the current case, can be attributed to reduced tissue perfusion following REBOA, the administration of relatively large amounts of contrast medium (400 ml at 300 mI), and increased intraperitoneal pressure. Nevertheless, the patient demonstrated significant recovery despite requiring temporary renal replacement therapy. A meta-analysis by Granieri et al. reported frequencies of acute kidney injury in REBOA insertion and control groups of 10% and 6%, respectively, among patients with blunt or penetrating traumatic torso hemorrhages and hemodynamic instability [11].

## Conclusions

Our case report demonstrates the effective management of hemodynamically unstable blunt splenic injury using REBOA and SAE with the early involvement of radiologists, highlighting the expanding role of endovascular management approaches in trauma cases. Nonetheless, utmost caution should be exercised during TAE when blood flow is not within normal physiological parameters.
